# Draft genomes of *Limimaricola soesokkakensis* TX01 isolated from the light organ of *Anomalops katoptron* in Indonesian waters

**DOI:** 10.1128/mra.00934-26

**Published:** 2026-09-03

**Authors:** Bo Yang, Yanping Zhao, Fangfang Zhang, Qi Jiang, Haiyue Yu, Zhiyong Yue

**Affiliations:** 1 Key Laboratory of Natural Anti-aging Product Mining and Biosynthesis of Shaanxi Higher Education Institutes, College of Medicine, Xi'an International University 467045, Xi'An, Shaanxi, China; Montana State University, Bozeman, Montana, USA

**Keywords:** *Limimaricola soesokkakensis*, genome analysis

## Abstract

The draft genome sequence of *Limimaricola soesokkakensis* TX01, isolated from the light organ of *Anomalops katoptron*, which lives in the region of the Banda Islands, Indonesia. The assembled genome is 4,041,441 base pairs in length, distributed across 69 contigs, with a GC content of 67.21%, and encodes 3,823 predicted protein-coding genes.

## ANNOUNCEMENT

*Anomalops katoptron* inhabits Indonesian archipelagos in the Pacific Ocean and possesses specialized light organs (1). The well-characterized luminous symbiont responsible for its bioluminescence is *Candidatus Photodesmus katoptron* (2). Aside from this obligate luminous symbiont, the present study aimed to isolate and characterize other non-luminous bacterial taxa residing within the light-organ, to explore potential genetic mechanisms underlying bacterium-fish symbiotic interactions in future work.

Samples of *A. katoptron*, which were initially captured near Banda Island, Indonesia (4°31′30″S, 129°52′17″E), were obtained from a licensed ornamental-fish importer of China. The light organs were excised from individual fish specimens, rinsed repeatedly with sterile distilled water, and surface-sterilized via immersion in 75% ethanol for 3 min. Following surface disinfection, the light organs were homogenized aseptically. The resulting tissue homogenate was serially diluted using sterile liquid 2216E medium (Difco, USA) (3), spread onto 2216E marine agar plates, and incubated at 28 °C for 120 h. Single bacterial colonies were picked from the agar plates and purified through repeated streaking subcultures.

For genomic DNA extraction, the strain was cultured in liquid 2216E medium (3). Incubation conditions were set at 28°C with shaking at 200 rpm in 250 mL shake flasks, and the duration of incubation was 72 h. The bacterial pellets were collected via centrifugation. Genomic DNA was then extracted from cell pellets using the CTAB method (4). The concentration and purity of purified genomic DNA were quantified and validated with a NanoDrop spectrophotometer (Thermo Scientific, USA) and Qubit 4.0 Fluorometer (Thermo Scientific, USA) to assess DNA quality.

**Fig 1:**
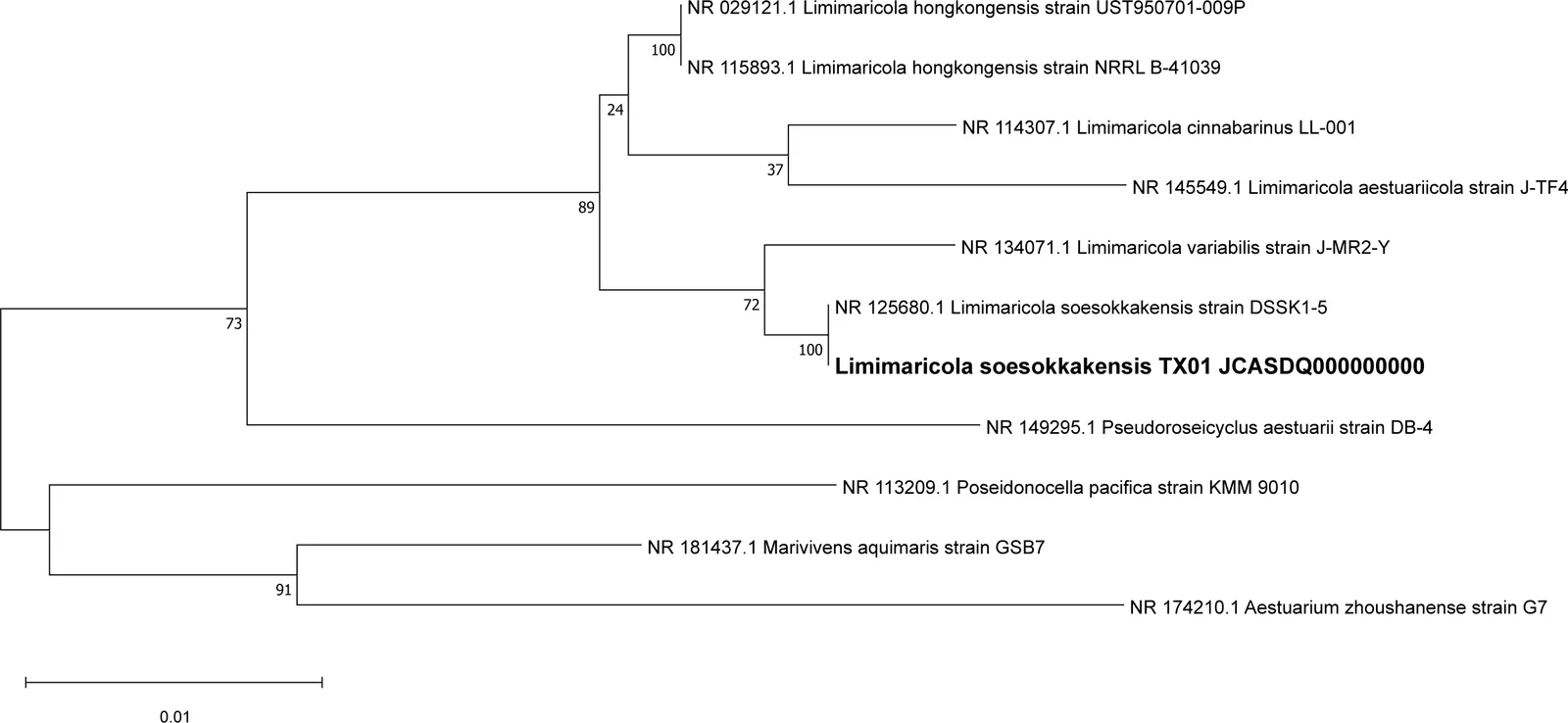
Maximum-likelihood phylogenomic tree inferred from the 16S rRNA gene sequences of 11 Rhodobacterales. The sequences were from the NCBI database. The substitution model of Tamura-Nei was selected, and branch support was estimated using 1,000 bootstrap replicates. The scale bar represents substitutions per site.

The 16S ribosomal RNA (rRNA) gene was amplified using the 27F/1492R primer pair (5). Phylogenetic analysis based on 16S rRNA sequences identified the isolate as *Limimaricola soesokkakensis* (Fig. 1), designated strain TX01 herein. Whole-genome sequencing was conducted on the MGISEQ-2000 platform (MGI Tech, China) with 2 × 150 bp paired-end reads. Raw sequencing reads were preprocessed via TrimGalore (v0.6.7) (6) to remove sequencing adapters, trim low-quality bases, and filter low-quality reads. Clean reads were subsequently normalized and error-corrected with BBNorm (v39.91) (7). Genome assembly was completed using Unicycler (v0.4.8) (8), a pipeline that automatically integrates Bowtie2 (v2.4.4) (9) for read mapping; Samtools (v1.20) (10) to sort, index, and process alignment files; and Pilon (v1.24) (11) for iterative polishing to fix single-base mismatches and small indels within assembled contigs. QUAST (v5.2.0) (12) was applied to thoroughly assess assembly quality through calculation of quantitative metrics: total assembly size, contig count, largest contig length, N50, N90, and GC content. The final assembled genome was uploaded to the RAST web server (13) for preliminary genomic feature characterization and functional genome annotation. All bioinformatic software was executed with default parameters unless stated otherwise.

The final draft genome of *L. soesokkakensis* TX01 spans 4,041,441 base pairs and consists of 69 contigs, with an average sequencing depth of 268× and a GC content of 67.21%. Genome annotation with the National Center for Biotechnology Information (NCBI) Prokaryotic Genome Annotation Pipeline v6.11 (14) predicted a total of 3,916 genes, of which 3,823 are protein-coding genes, 3 rRNAs, 45 transfer RNAs (tRNAs), and 45 others (Table 1).

**TABLE 1:** Genomic features of *Limimaricola soesokkakensis* TX01

Genomic characteristic	Value
Total assembly size (bp)	4,041,441
Number of contigs	69
Largest contig length (bp)	512,438
N50	218,465
N90	52,575
GC content (%)	67.21
Sequencing depth (×)	268
Total genes	3,916
Protein-coding genes	3,823
rRNA genes	3
tRNA genes	45

## Data Availability

This whole-genome sequence has been deposited in DDBJ/ENA/GenBank with accession number JCASDQ000000000.1 under BioProject accession number PRJNA1490922 and BioSample accession number SAMN61439104. The accession number of the publicly available raw data of the genome sequence is SRR39867025.
